# A method to study and enhance the energy efficiency of soft electrostatic actuators

**DOI:** 10.1073/pnas.2527676123

**Published:** 2026-02-06

**Authors:** Steven L. Zhang, Toshihiko Fukushima, Sophie Kirkman, Soo Jin Adrian Koh, Philipp Rothemund, Christoph Keplinger

**Affiliations:** ^a^Robotic Materials Department, Max Planck Institute for Intelligent Systems, Stuttgart 70569, Germany; ^b^Institute for Adaptive Mechanical Systems, University of Stuttgart, Stuttgart 70569, Germany; ^c^Paul M. Rady Department of Mechanical Engineering, University of Colorado, Boulder, CO 80309; ^d^Materials Science and Engineering Program, University of Colorado, Boulder, CO 80309

**Keywords:** soft robotics, actuators, energy efficiency, electrostatic transducers, HASEL actuators

## Abstract

Actuators drive robotic motion, and their energy conversion efficiency is a key performance metric that informs power consumption. Soft electrostatic actuators promise new opportunities for bioinspired and wearable robotics, being driven by electrical signals and producing high-speed, muscle-like motion. Unlike electromagnetic motors, for which efficiency has been systematically studied, efficiency of soft actuators lacks a standardized definition and measurement method, highlighting the need for a unified framework for the evaluation of their efficiency. Here, we propose a comprehensive method to study electrical-to-mechanical energy conversion in soft electrostatic actuators by analyzing closed cycles on planes spanned by work-conjugate variables: voltage–charge and force–position; our experimental setup allows us to prescribe and measure in real-time all work-conjugate variables and thus, to evaluate efficiency as function of load, electric potential, frequency, and actuator materials. We introduce a practical work cycle to evaluate actuators, and, using Peano-HASEL (Hydraulically Amplified Self-healing ELectrostatic) actuators as a model system, we reveal that efficiency is highly dependent on applied voltage, force, and actuation frequency; within the tested range of parameters, we measure a maximum efficiency of 63.6%, which is more than three times the previously reported value for HASEL actuators. We further study energy losses inherent in mechanical and electrical cycles. We show the general applicability of our method across different electrostatic actuators by applying it to a pure-shear dielectric elastomer actuator (DEA), demonstrating efficiencies up to 62.9%. This comprehensive method will facilitate the study and development of electrostatic actuators for the next generation of highly efficient soft robots.

## Significance

The electrical-to-mechanical energy conversion efficiency of actuators is a key metric, which determines the energy consumption of robotic devices. In the field of soft electrostatic actuators, there is currently no universally agreed-upon way to calculate and measure the efficiency of actuators. This article addresses this gap by introducing a comprehensive method to measure and optimize the efficiency of soft electrostatic actuators, thereby proposing a common framework for efficiency measurement that facilitates the development of highly efficient soft robotic systems.

Conventional robotic systems—relying on electromagnetic motors—perform repetitive tasks accurately, reliably, and precisely. However, they face challenges when deployed in unstructured environments (1–4). Soft actuators—made of flexible or stretchable materials (5)—feature inherent embodied intelligence (6), which allows them to adapt to unstructured environments naturally (2). Soft actuators are driven by a variety of stimuli, such as fluid pressure (7–9), electric field (10–12), magnetic field (13, 14), temperature (15, 16), and chemical potential (17, 18). Soft electrostatic actuators, such as dielectric elastomer actuators (DEAs) (19–22), and those that exploit liquid dielectrics (23, 24), like Hydraulically Amplified Self-healing ELectrostatic (HASEL) actuators (25–29), exhibit muscle-like performance. They are driven directly by electrical signals and could therefore use miniature power supplies (30, 31), facilitating the design of agile, untethered robots (32, 33) and wearable robotic systems (34, 35).

Energy conversion efficiency is a key performance metric for actuators, as it impacts the energy consumption of robots and thus their operating time when they are untethered (36). Studies of the efficiency of electromagnetic motors have revealed that conversion efficiency is not a single-valued metric but instead varies considerably, depending on operational parameters like current, torque, and speed (37). Standardized performance curves provide data to maximize the operational efficiency of electromagnetic motors, to evaluate and optimize the overall efficiency of motor-powered systems, and to facilitate efficiency comparison across different motor designs.

In contrast, the energy conversion efficiency of soft electrostatic actuators has not been comprehensively studied—often, it is only measured for one set of operating parameters and is reported as a single value. The reported values are seen as representative of an entire class of actuators, for example, 21% for HASEL actuators (28), even though efficiencies may vary drastically across operating conditions, materials systems, and actuator designs. Importantly, there is no universally agreed-upon way to determine the efficiency of soft electrostatic actuators. While efficiency is commonly understood to be the ratio of mechanical work output to electrical energy input, it is measured in fundamentally different ways. We and others used closed work cycles on both the mechanical and electrical work-conjugate planes to measure efficiency (26, 28, 38, 39). In contrast, some works only considered the parts of work cycles in which electric energy is added to an actuator and work is done against a load (40, 41). Kornbluh et al. inferred efficiency from electromechanical coupling constants, analogous to the field of piezoelectrics (42, 43). Researchers have also used mechanical and dielectric loss tangents (44–46) as well as the kinetic energy transferred to an object (19) to infer the electromechanical efficiency. Overall, we note that the community lacks both a deeper understanding of how efficiency varies across design and operating parameters, as well as a unified way to define and measure energy conversion efficiency; these shortcomings prevent meaningful comparisons of values reported in different works and hamper the further development of more efficient soft electrostatic actuators.

Here, we introduce a comprehensive method to study the electrical-to-mechanical energy conversion efficiency in soft electrostatic actuators, by measuring and analyzing closed work cycles on planes spanned by the work-conjugate variables of voltage–charge, and force–position; we present an experimental setup that allows us to prescribe and measure all work-conjugate variables and systematically vary experimental parameters such as force, voltage, frequency, and actuator materials. We propose a practical work cycle and investigate the efficiency of a model actuator—the Peano-HASEL actuator—and show that efficiency is highly dependent on the applied force and voltage, cycle frequency, and actuator materials. Within the tested experimental conditions, we measured a maximum efficiency of 63.6%, three times higher than the previously reported value for HASEL actuators (26, 28). Additionally, we prescribed closed cycles with zero electrical energy input and with zero mechanical work output to investigate the inherent mechanical and electrical losses, respectively, which reduce the closed-cycle efficiency of Peano-HASEL actuators. To demonstrate that our method is applicable to other soft electrostatic actuators, we use it to measure the efficiency of a pure-shear DEA (38), for which we obtain a maximum efficiency of 62.9%; consequently, the method may also be readily applied to electroribbon actuators (24), electrostatic bellows actuators (23), and electrohydrodynamic pumps (47, 48), and it may serve as a standard for the characterization, comparison, and optimization of the energy conversion efficiencies of different types of soft electrostatic actuators.

## Results

**Fig. 1.**
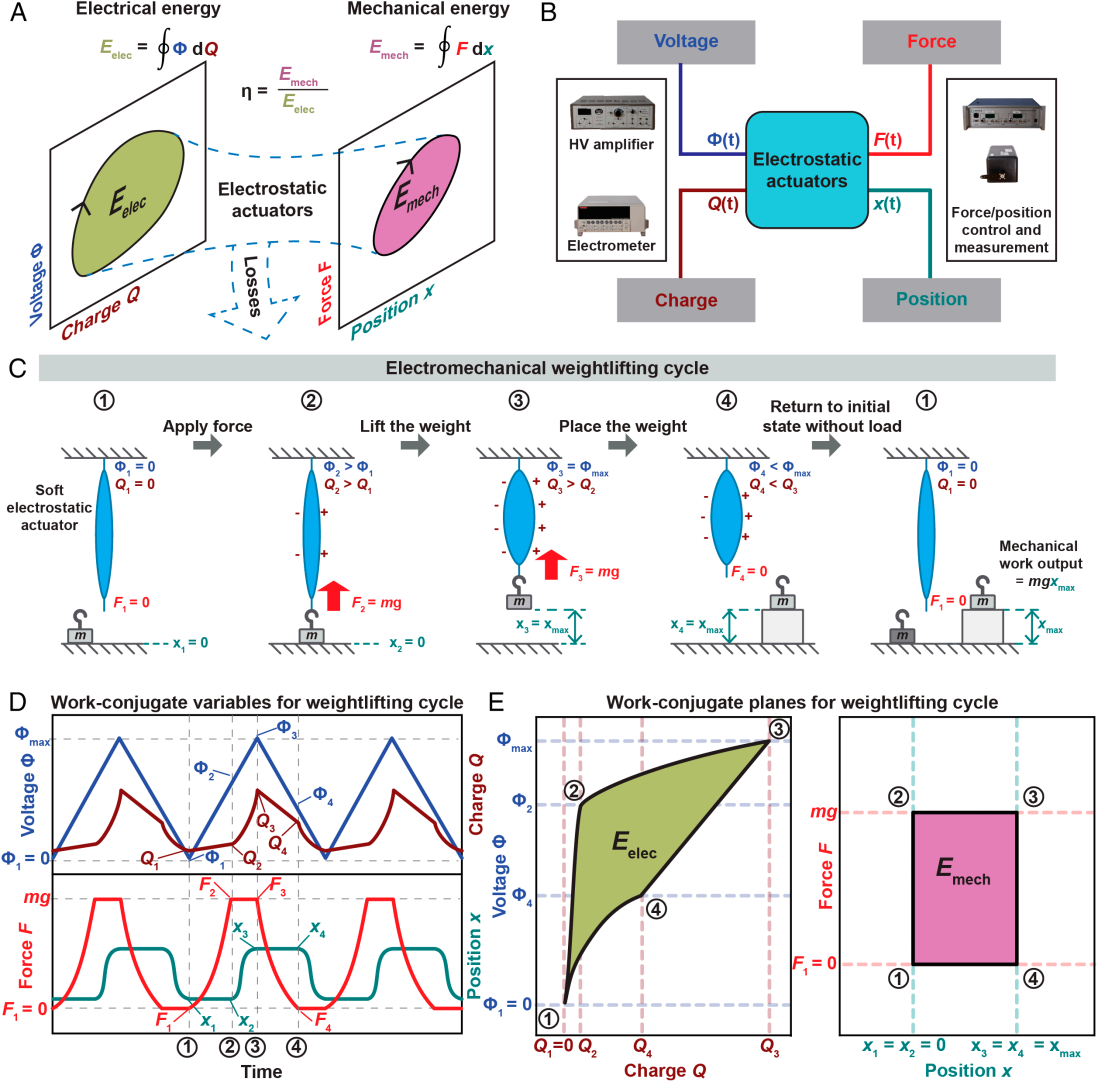
Evaluation of energy conversion efficiency of soft electrostatic actuators in planes spanned by work-conjugate variables. (*A*) The states of electrostatic actuators can be represented as points on the voltage–charge and force–position work-conjugate planes. A work cycle forms closed loops on these planes. The enclosed areas on the voltage–charge and force–position planes correspond to the electrical energy input and the mechanical work output of the cycle, respectively. The efficiency is the ratio of the mechanical work output and the electrical work input. (*B*) Experimental characterization of the efficiency requires instrumentation to measure or prescribe the four state variables: voltage, charge, force, and position. (*C*) An example of a closed work cycle of a generic contractile soft electrostatic actuator that transports a weight to an elevated position and returns to its original state. (*D*) Schematic of the behavior of voltage, charge, force, and position of this cycle as a function of time, and (*E*) the voltage–charge and force–position planes for the electromechanical weightlifting cycle (20, 43).

The equilibrium state of an electrostatic actuator is defined by the work-conjugate state variables voltage Φ and charge *Q*—which describe the electrical state of the actuator—and force *F* and position *x*—which describe the mechanical state of the actuator. These variables span two work-conjugate planes: the voltage–charge and the force–position planes, on which an equilibrium state is represented as a point (Fig. 1*A*) (49). During actuation, the state of the actuator moves along a path on each of these two planes, and a closed work cycle will form a closed loop. For an actuator, the area enclosed by the loop on the voltage–charge plane corresponds to the input electrical energy (*E*_elec_), and the area enclosed by the loop on the force–position plane corresponds to the output mechanical work (*E*_mech_). The difference between these areas corresponds to the energy lost over the full cycle. Analogous to the definition of thermal conversion efficiency in thermodynamics (50), we define the electrical-to-mechanical conversion efficiency (η) of an electrostatic actuator as:

[1] $$\eta =E_{\mathrm{mech}}/E_{\mathrm{elec}}.$$

The work cycle of an electrostatic actuator is fully defined by prescribing the time evolution of two of the four state variables; the remaining two are dependent variables, which are measured (51). Thus, the experimental characterization of the efficiency of an electrostatic actuator requires an experimental setup that can prescribe two of the state variables and measure the remaining two (Fig. 1*B*). An example of a simple work cycle for a soft electrostatic actuator (Fig. 1 *C* and *D*), termed an “electromechanical weightlifting cycle,” is described as follows: The actuator, initially unloaded and unactuated, lifts a weight from a low to a high elevation, deposits the weight there, and then returns to its initial position and state. This last step in the cycle, the return to the initial position, is important to include in efficiency considerations, as this step typically causes additional energy flows. The resulting enclosed area on the voltage–charge plane corresponds to the electrical energy expended during the cycle, and the enclosed area on the force–position plane corresponds to the mechanical work done to lift the weight (Fig. 1*E*). Importantly, cycles where the weight merely moves up and down with the actuator, without the weight being deposited at the elevated position, do not enclose any area in the mechanical work-conjugate plane (as it would be characterized by an oscillation between states 2 and 3 in Fig. 1*E*), which means that no mechanical work was done and the efficiency is zero.

**Fig. 2.**
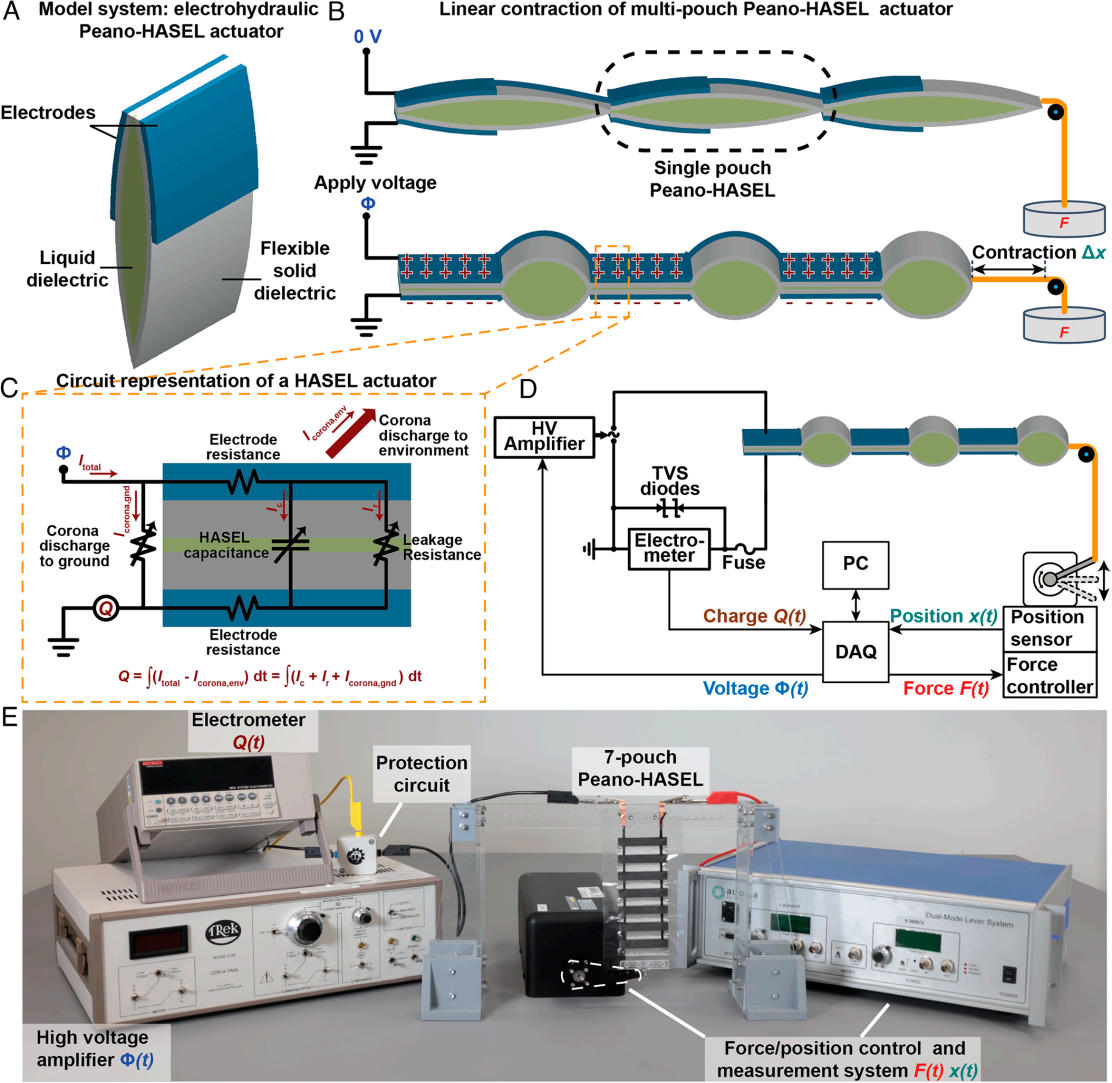
Principles of Peano-HASEL actuators, used as a model system for soft electrostatic actuators, and experimental setup to measure energy conversion efficiency. (*A*) A Peano-HASEL actuator consists of a rectangular, inextensible, and flexible solid dielectric shell, that is partially coated on both sides by flexible electrodes and filled with liquid dielectric. (*B*) Multiple (here, three are shown) Peano-HASEL actuators can be connected in series to increase stroke. When a voltage Φ is applied across the electrodes, charge *Q* flows onto them. The actuator contracts by Δ*x* to lift a load *F*. (*C*) Circuit representation of a Peano-HASEL actuator, and (*D*) our experimental setup to characterize the efficiency of soft electrostatic actuators for force and voltage control. (*E*) Photograph showing the experimental setup in (*D*).

To demonstrate efficiency measurements with closed work cycles, we use the Peano-HASEL actuator as a model system for the class of soft electrostatic actuators. Peano-HASEL actuators are made by sealing two flexible but inextensible dielectric films into a rectangular pouch (25, 27, 52) (*Materials and Methods*). The top half of each side of the pouch is coated with flexible electrodes, and the pouch is filled with liquid dielectric (Fig. 2*A*). Multiple pouches are connected in series to scale up the actuation stroke (Fig. 2*B*). When a high voltage is applied between the electrodes, charges of opposite polarity flow onto the electrodes (Fig. 2*B*), which then, by virtue of the Maxwell stress generated between the opposite charges, cause the electroded films to close the liquid gap in a zipping motion, and push the liquid out from between the electrodes, causing the actuator to contract. This contraction generates a force that lifts a weight and thus performs mechanical work. When the applied voltage is reduced, the actuator unzips and lengthens. From an electrical perspective, HASEL actuators are deformation-dependent variable capacitors with various possible sources of electrical loss. Both dielectric materials have finite resistances and allow electrical charges to leak through them (Fig. 2*C*). Furthermore, the electrode resistance dissipates electrical energy through resistive heating. Additionally, high electrical potential on the high voltage electrode ionizes its surroundings, manifested as corona discharge near the edges of the electrodes, creating a discharge path for charges to leak into the environment, some of which can migrate around the actuator back into the ground electrode.

**Fig. 3.**
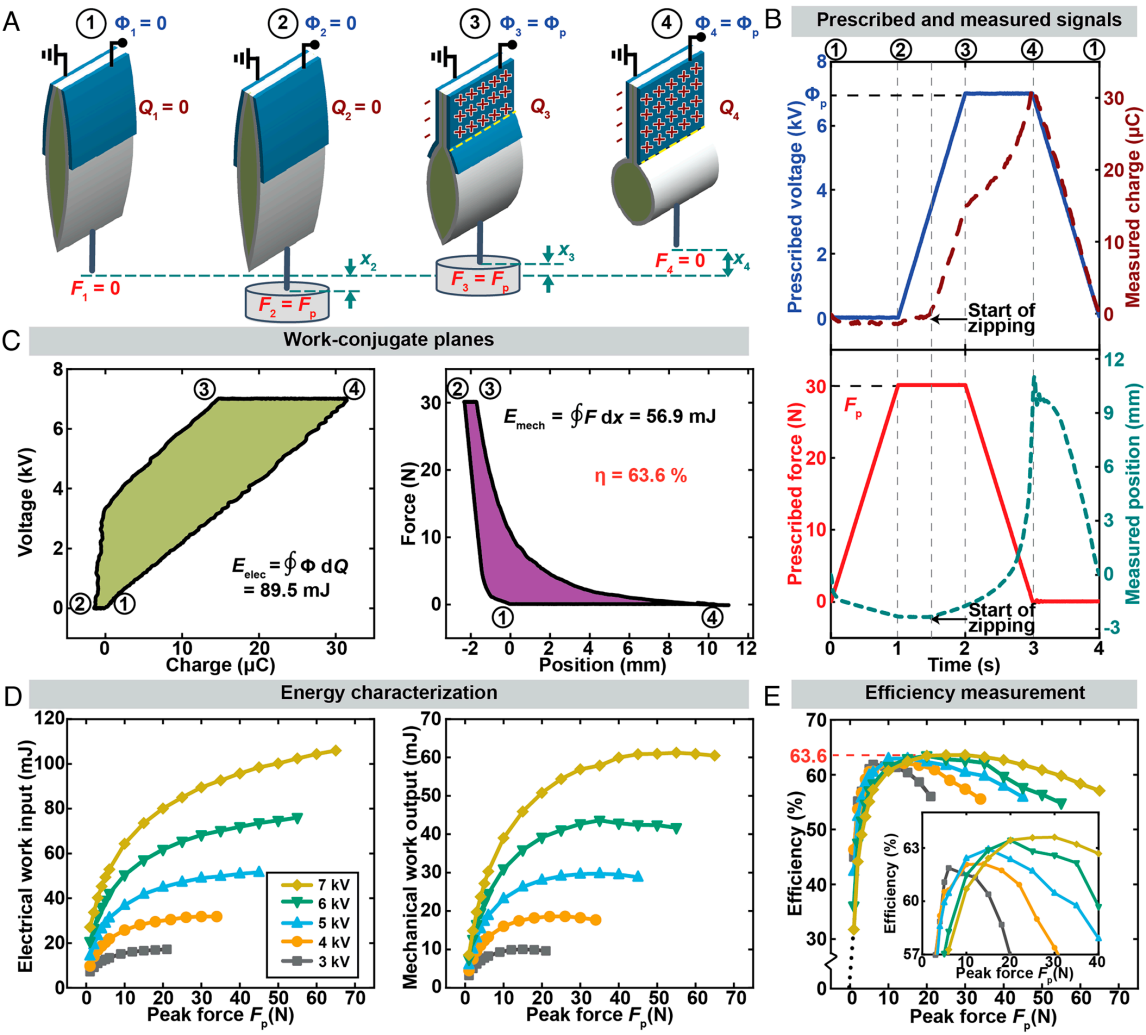
Closed-cycle efficiency measurement of a 7-pouch Peano-HASEL actuator as function of force and voltage. (*A*) Illustration of the chosen test cycle, shown for 1 of the 7 pouches of the Peano-HASEL actuator. (*B*) Experimentally prescribed and measured state variables as a function of time shown for the conditions with the highest efficiency of 63.6% (Φ_p_ = 7 kV, *F*_p_ = 30 N). (*C*) The corresponding work cycle on the voltage–charge and the force–position planes. The enclosed areas correspond to the electrical energy input and the mechanical work output. (*D*) Measured electrical energy input and measured mechanical work output as a function of the prescribed voltage and the prescribed force. (*E*) Electromechanical conversion efficiency as a function of the prescribed voltage and the prescribed force. Inset shows that for each peak force, there is an optimum peak voltage (*SI Appendix*, Fig. S7). The shells of the actuators used in this figure were made from 15-μm polyethylene terephthalate (PET) (Mylar). All data were measured at a cycle frequency of 0.25 Hz.

We introduce an experimental setup to measure the electrical-to-mechanical energy conversion efficiency of soft electrostatic actuators, which allows us to prescribe arbitrary work cycles and to systematically vary the operational parameters (Fig. 2 *D* and *E*; see *SI Appendix* for details on devices). An electrometer measures the charges that flow from ground onto the actuator, allowing it to collectively measure charges stored on the actuator, charges that have leaked through it, and the corona discharge that flows around the actuator and returns to ground (*SI Appendix*, Figs. S1 and S2); it does not explicitly measure the corona discharge to the environment, as indicated in the equation shown in Fig. 2*C*. The electrometer is more accurate than current-based methods for charge measurements (26, 28, 39) because it performs a direct coulombic charge measurement and thus, avoids integration errors that arise when charge is inferred from current signals. The electrometer is protected from overvoltage and overcurrent by a custom protection circuit (see *SI Appendix* for components). Mechanical load is applied with a dual-mode lever system which allows the application of arbitrary load profiles using either position or force control and thus provides more accuracy, flexibility, and repeatability than manual methods to apply mechanical load (e.g., adding and removing weight) (28). A high voltage amplifier capable of operating in voltage control and charge control modes is used to apply the electrical load to the actuator. A data acquisition (DAQ) system is used to output the driving signals and to record the input signals synchronously. This experimental setup facilitates the characterization of efficiency under different combinations of prescribed and measured variables, for arbitrary pairs of voltage/charge and force/position (Fig. 3 and *SI Appendix*, Fig. S3). In this study, we used Peano-HASEL actuators consisting of seven pouches connected in series. We attached rigid mounts to the top and bottom of the actuators; the top mount is fixed to a rigid stand and the bottom mount is directly connected to the lever system to minimize parasitic mechanical losses, which always arise from loose connections.

While the previously introduced weightlifting cycle (Fig. 1*C*) is simple to understand, it is complex to implement experimentally with the dual-mode lever system as it is necessary to switch from force control to position control mid-cycle, requiring an intricate feedback loop for precise transition. We hence adopted an easier-to-implement cycle that only requires force-control and is defined by the peak applied voltage (Φ_p_) and the peak applied force (*F*_p_) (Fig. 3 *A*–*C*). This electromechanical cycle is analogous to the thermodynamic Ericsson cycle, where heat is converted into mechanical work through a controlled cyclic process involving isothermal and isobaric transformations, which are, equivalently, the constant voltage and constant force processes in our experimental cycle (53). Fig. 3*A* and *SI Appendix*, Fig. S4 illustrate snapshots of a single-pouch Peano-HASEL actuator undergoing this cycle. In the initial state (state 1), no voltage and no load are applied to the actuator (Φ_1_ = 0, *F*_1_ = 0). Consequently, the electrodes have zero electrical charge (*Q*_1_ = 0), and the actuator is at its undeformed position (*x*_1_ = 0). From state 1 to state 2, the force is gradually increased to a force *F*_2_ = *F*_p_, while no voltage is applied (Φ_2_ = 0); the actuator deforms to a position *x*_2_ but remains uncharged (*Q*_2_ = 0). From state 2 to state 3, the applied voltage is ramped up linearly to Φ_3_ = Φ_p_, while the force is held constant (*F*_3_ = *F*_p_); charges *Q*_3_ flow onto the electrodes, and the actuator contracts to a position *x*_3_. From state 3 to state 4, the applied force is gradually unloaded to the initial force *F*_4_ = 0, while the voltage is held constant (Φ_4_ = Φ_p_); as a result, the number of charges on the electrodes increases to *Q*_4,_ and the actuator further contracts to a position *x*_4_. Mechanical work output occurs while transitioning from states 2 to 4. From state 4 to state 1, the applied voltage is reduced back to Φ_1_ = 0; the actuator discharges so that the actuator returns to approximately its initial position *x*_1_ = 0.

Using the cycle as described above, we characterized the efficiency of a 7-pouch Peano-HASEL actuator made with 15-μm-thick Mylar as the shell material (Fig. 3 *A*–*C* and *SI Appendix*, Fig. S4 and Movie S1). We used a 4-s cycle period (0.25 Hz) consisting of four one-second segments of prescribed voltage and force ramps and holds (Fig. 3*B*). We cycled the actuator ten times before recording data to allow it to attain steady-state (*SI Appendix*, Fig. S5). We used work-conjugate planes shown in *SI Appendix*, Fig. S6 to calculate the electrical energy input and the mechanical work output (Fig. 3*D*) as a function of prescribed peak voltages Φ_p_ ranging from 3 kV to 7 kV (beyond which, dielectric breakdown is more likely to occur), and as a function of prescribed peak forces *F*_p_ ranging from 0 N to 65 N (beyond which we observed significant plastic deformation in the actuator). The measured electrical energy input increased with both the prescribed peak voltage and load. The measured mechanical work output increased with the applied peak voltage due to the resulting larger actuation strains. For each prescribed voltage, the mechanical work output increased and then decreased with increasing prescribed peak force.

We calculated the closed-cycle efficiency of the actuators using Eq. **1** (Fig. 3*E*). The efficiency was highly dependent on the operating conditions, indicating that for robotic applications, the operating parameters should be chosen carefully to enable efficient operation. For each given peak voltage, there is an optimum peak force (which results in the highest efficiency); and for each given peak force, there exists an optimum peak voltage (*SI Appendix*, Fig. S7). For our cycle, the maximum efficiency of 63.6% occurred at a prescribed peak voltage of 7 kV and a prescribed peak force of 30 N. This number is triple the previously reported values for HASEL actuators—21% for a thermoplastic quadrant donut HASEL (28) and 19% for an elastomeric donut HASEL (26)—and also larger than the efficiency of human muscles [~40% (54)]. The key reason for this increase from previously reported efficiency values for HASELs is that only one specific set of operating conditions was used for efficiency measurement in the previous works; in particular, a combination of a low applied force (1 N) and a high voltage (up to 21 kV) (26, 28) was used, which is likely far from the optimum conditions for the tested actuator. We also tested Peano-HASELs with different pouch widths to explore how geometry influences efficiency and optimum operating force. At constant voltage, the force at which the efficiency peaked was proportional to the width of the actuator (*SI Appendix*, Fig. S8), which agrees with previous results indicating that normalized performance metrics of Peano-HASEL actuators are independent of actuator width (52, 55).

**Fig. 4.**
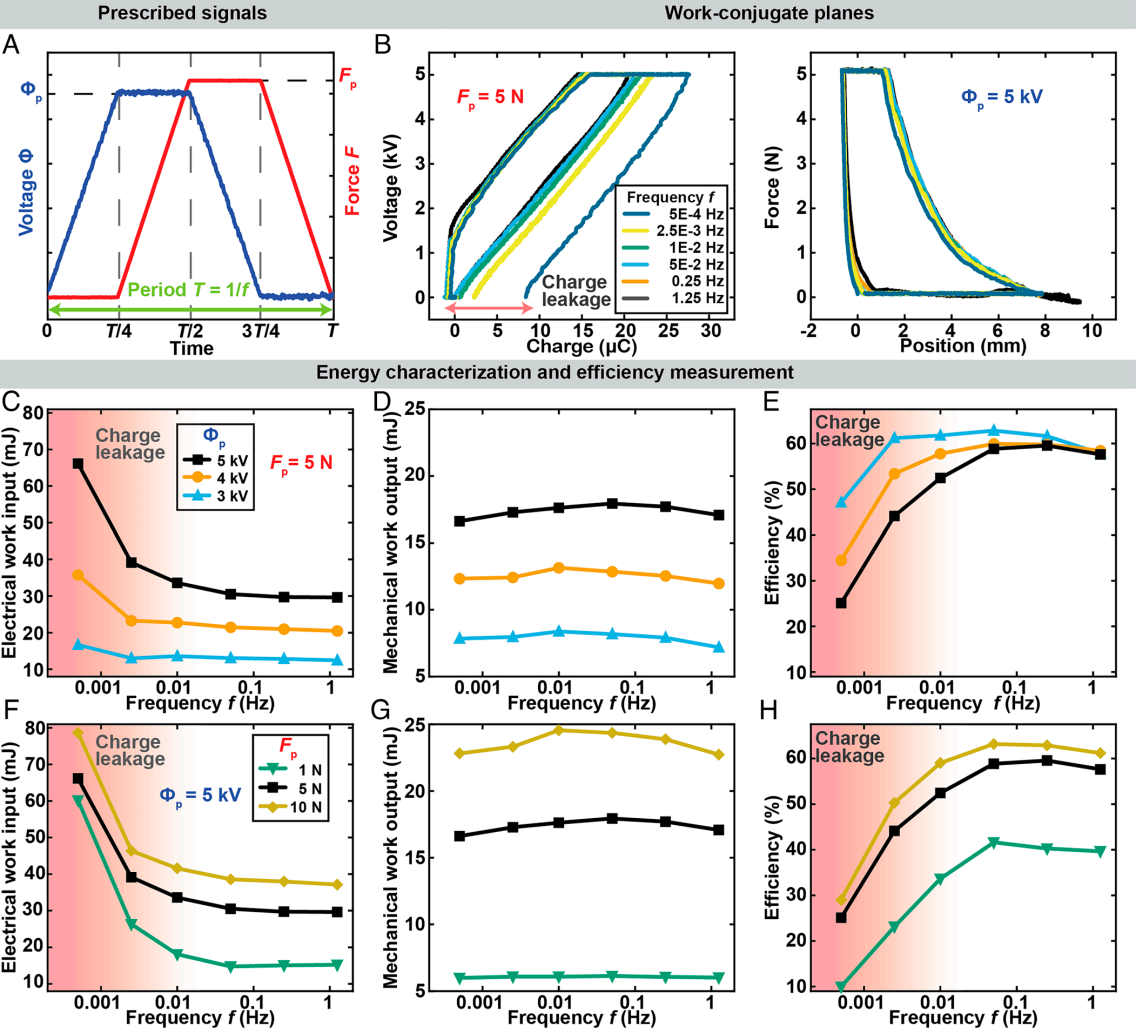
Characterization of the effect of frequency on closed-cycle efficiency. (*A*) Prescribed voltage and force as function of time. (*B*) Work cycles measured at different frequencies. For frequencies of 0.5 mHz and 2.5 mHz, the cycle did not close due to charge leakage. (*C*) Electrical work input, (*D*) mechanical work output, and (*E*) the resulting efficiency as a function of frequency at the same peak force *F*_p_ for different peak voltages Φ_p_. The shaded region indicates the frequency range where charge leakage reduces efficiency. (*F*) Electrical work input, (*G*) mechanical work output, and (*H*) efficiency as a function of frequency at the same peak voltage Φ_p_ for different peak forces *F*_p_.

Actuators operate at a range of different speeds for different applications (52). Hence, it is also relevant to study the effect of cycle frequency on efficiency. We, thus, varied the cycle period, *T,* while keeping the prescribed peak voltage (5 kV) and peak force (5 N) constant (Fig. 4*A*). We tested cycle frequencies spanning from 0.5 mHz to 1.25 Hz, where the upper limit was determined by the speed of the used dual-mode lever system under force control. To achieve higher frequencies, position control was implemented to measure efficiency at up to 5 Hz (*SI Appendix*, Fig. S9), revealing that efficiency decreases above 1 Hz due to decreasing work output of the actuator at higher frequencies. Fig. 4*B* presents the cycle paths plotted on the work-conjugate voltage–charge and force–position planes. Substantial charge leakage was recorded in the millihertz regime, manifested by an open cycle in the voltage–charge work-conjugate plane. We attribute these leaked charges to conductive leakage through the film and oil, and to corona discharge from the high voltage electrode to the ground (Fig. 2*C*). The magnitude of charges leaked was computed by the size of the open loop (*SI Appendix*, Fig. S10), indicating that in the low frequency regime, a larger electrical energy input is required for the same amount of mechanical work to compensate for charge leakage losses (Fig. 4 *C*, *D, F*, and *G*), thereby reducing the efficiency (Fig. 4 *E* and *H*).

### Effect of Different Solid Dielectrics on Efficiency.

**Fig. 5.**
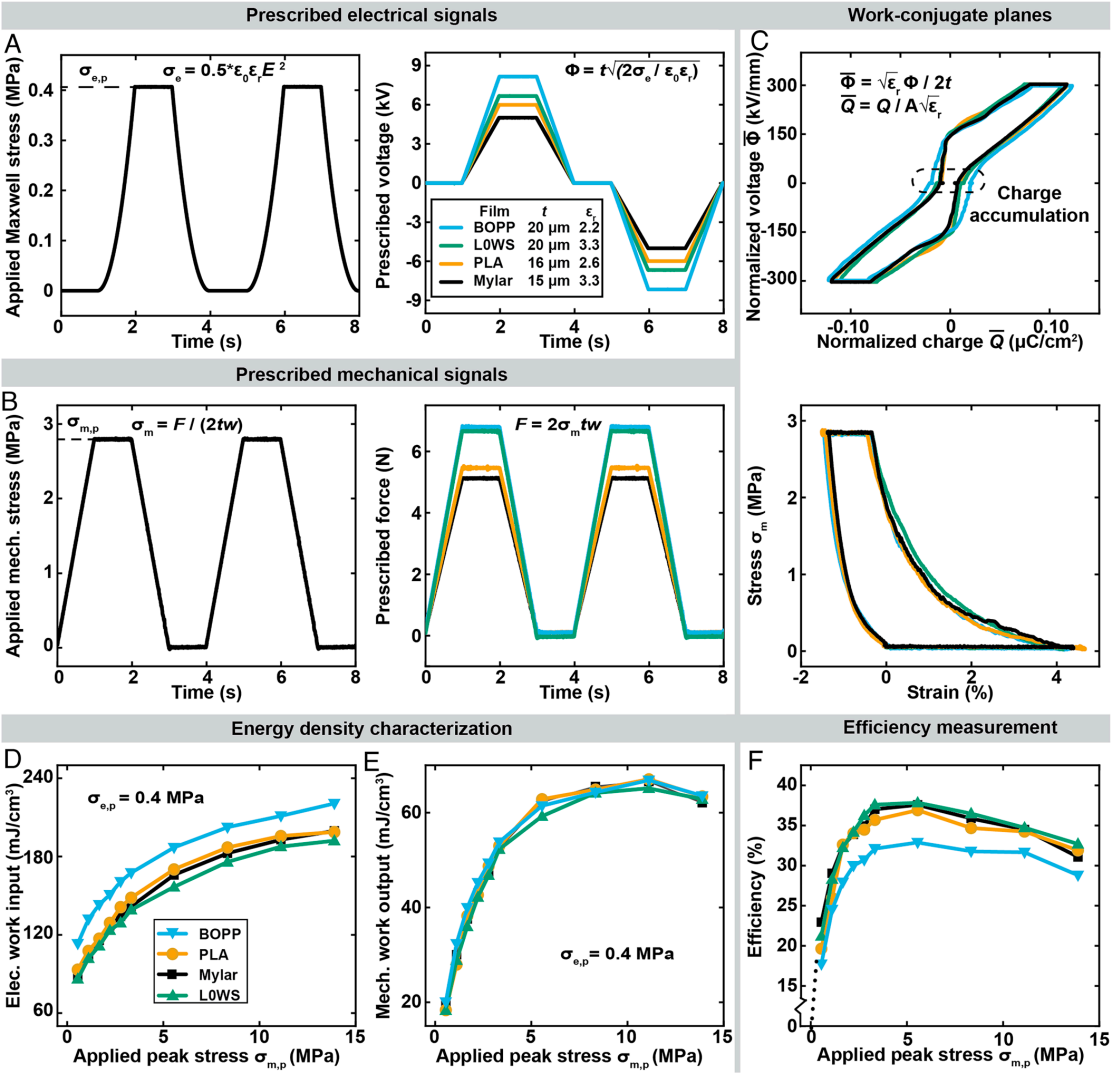
Characterization of the effect of different materials on the closed-cycle efficiency. (*A*) Maxwell stress in the zipped-region of the actuator as a function of time and the prescribed voltages to generate this Maxwell stress in actuators made of different materials. (*B*) Mechanical stress applied to the actuators as a function of time and the prescribed forces to generate this mechanical stress. (*C*) Normalized voltage–charge plane and stress–strain plane, in which the work cycles of different materials are expected to be the same. (*D*) Electrical work input, (*E*) mechanical work output, and (*F*) efficiency as a function of the applied peak stress.

We characterized the effect of different solid dielectric materials on closed-cycle efficiency. In addition to Mylar, we tested 3 more commercially available films, PLA, L0Ws, and BOPP (*Materials and Methods* and *SI Appendix*, Fig. S11). Because these materials have different permittivity and thickness values, we normalized the prescribed voltage and force profiles to ensure a consistent actuation strain across all materials. By keeping the Maxwell stress the same, we achieve an equivalent actuation stress for each material, following the equations shown in Fig. 5 *A* and *B*. Due to interfacial charge accumulation, transient decay in actuation strain may occur as cycling progresses under a single-polarity voltage signal for some material combinations (56). Hence, we adopted reverse-polarity alternating voltages for all actuators so as to maintain constant actuation strokes over all cycles during test. To match the single-polarity cycle (Fig. 3), we used the same time of 1 s each for the linear voltage ramp and the linear force ramp.

We normalize the measured voltage–charge paths and force–position paths (*SI Appendix*, Fig. S12) so that they can be compared (Fig. 5*C*); the areas enclosed by the normalized voltage–charge and force–position paths correspond to a volumetric energy density (using the film volume). Three of the paths on the normalized voltage–charge plane, BOPLA, L0Ws, and Mylar gave nearly identical curves, whereas BOPP presents a larger hysteresis loop, primarily attributed to the wider girth at 0 V. This shows that BOPP requires higher electrical input, compared to the other materials (Fig. 5*D*). Since conductivity values of all tested films are within the same order of magnitude (*SI Appendix*, Fig. S11*C*), we expect them to have similar charge leakage magnitudes, based on the assumption that charge leakage is conduction-current dominated (Fig. 2*C*); therefore, we surmise that the larger electrical energy input in BOPP could be due to charge accumulated at the BOPP–oil interface. Since the actuators based on different materials have similar stress–strain curves (Fig. 5*C*), actuators made with the BOPP films exhibit a lower efficiency (Fig. 5 *E* and *F*).

### Measurement of Inherent Electrical and Inherent Mechanical Losses.

**Fig. 6.**
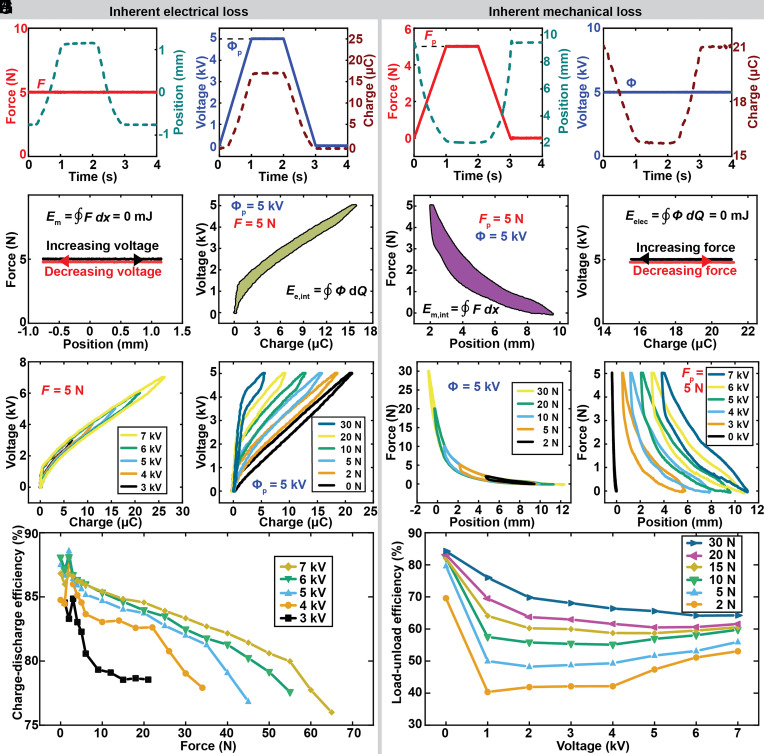
Cycles of net zero mechanical work and of net zero electrical energy to characterize inherent losses. (*A*) Prescribed and measured values for a net-zero mechanical work cycle. (*B*) Work-conjugate planes of inherent electrical loss cycle, showing net zero mechanical work. The area enclosed by the work cycle on the voltage–charge plane corresponds to the inherent electrical loss. Prescribed voltage with respect to measured charge for (*C*) different peak voltages, at a constant applied force of 5 N, and (*D*) with different constant applied forces at a peak voltage of 5 kV. (*E*) Charge-discharge efficiency as a function of force and peak voltage. (*F*) Prescribed and measured values for a net-zero electrical work cycle. (*G*) Work-conjugate planes of inherent mechanical loss cycle, showing net zero electrical work. The area enclosed by the work cycle on the force–position plane corresponds to the inherent mechanical loss. Prescribed force with respect to measured position for (*H*) different peak forces at a constant voltage of 5 kV and (*I*) different constant voltages with a peak force of 5 N. (*J*) Load-unload efficiency as a function of voltage and peak force.

To characterize inherent losses, we investigated cycles of net zero mechanical work and of net zero electrical energy, illustrated as horizontal time-invariant lines in Fig. 6 *A* and *F*. In these cycles, one work-conjugate variable is constant throughout the cycle, which means no area can be enclosed on the corresponding work-conjugate plane (Fig. 6 *B* and *G* and Movies S2 and S3). Consequently, these cycles yield an efficiency of either 0 (for net-zero mechanical work) or—mathematically—infinite (for net-zero electrical input). We measured the inherent electrical losses (Fig. 6 *C* and *D*) and inherent mechanical losses (Fig. 6 *H* and *I*) for various combinations of force and voltage.

Charge-discharge efficiency is a term widely studied for capacitors and batteries, and describes the ratio of recovered electrical energy to total electrical input energy (57) (*SI Appendix*, Fig. S13*A*). We observed higher charge-discharge efficiency at higher voltage and lower forces (Fig. 6*E*). In *SI Appendix*, Fig. S13*B*, we show absolute values for inherent electrical loss for the corresponding combinations of force and peak voltage; we observe higher inherent electrical losses for higher voltages and lower forces. Load-unload efficiency is commonly used to characterize hysteretic losses in rubber elasticity, and describes the ratio of recovered mechanical energy to total mechanical input energy (58) (*SI Appendix*, Fig. S13*C*). We observed higher load-unload efficiency at higher forces (Fig. 6*J*). In *SI Appendix*, Fig. S13*D*, we show absolute values for inherent mechanical loss for the corresponding combinations of voltage and peak force; we observe higher inherent mechanical losses for higher voltages and higher forces.

Overall, we see that the electrical charge-discharge efficiency is higher than the mechanical load-unload efficiency (Fig. 6 *E* and *J*).

### Measurement of Efficiency of DEAs.

**Fig. 7.**
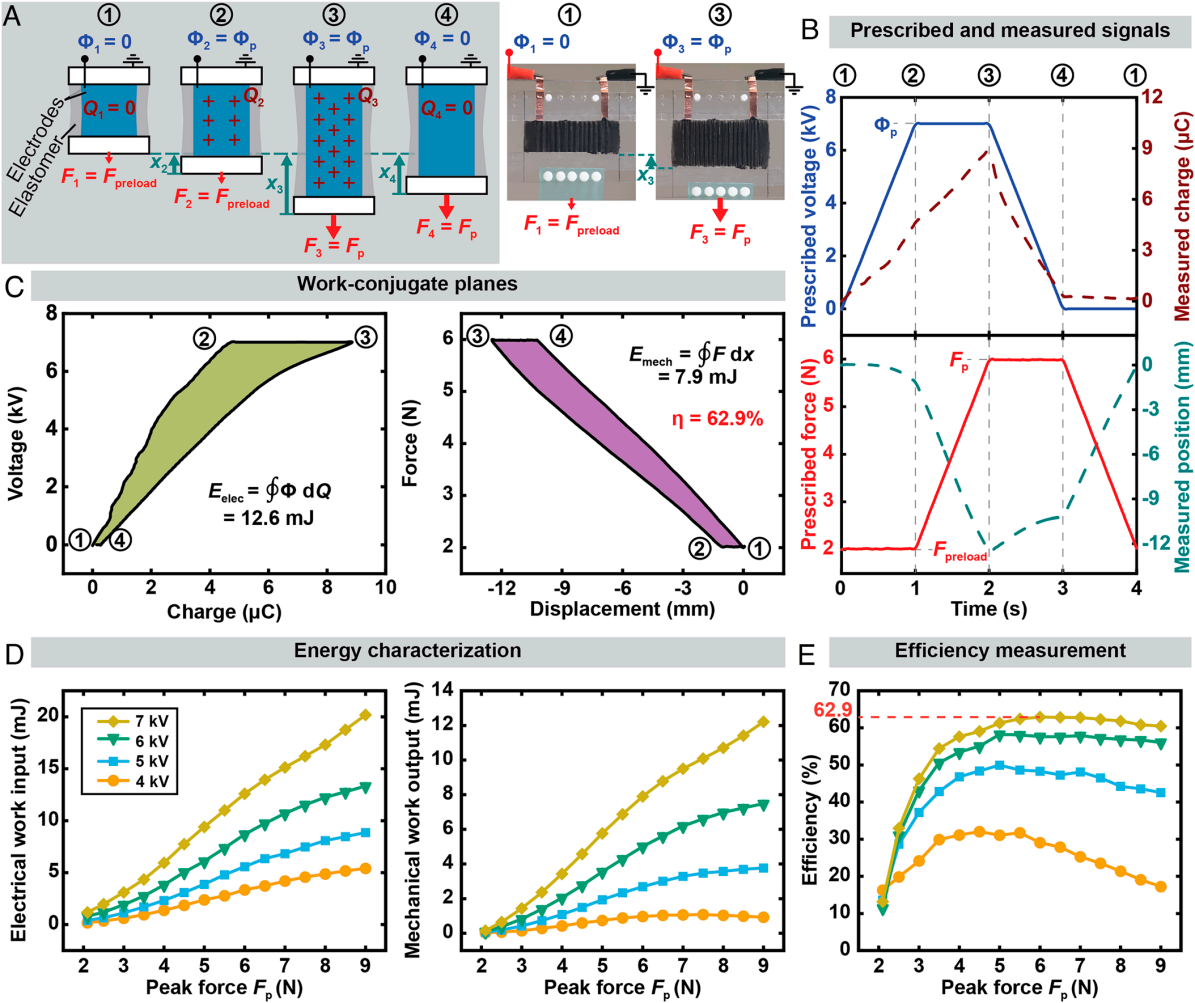
Closed-cycle efficiency measurement of a dielectric elastomer actuator (DEA) made from Elastosil (Wacker). (*A*) Illustration of the test cycle for a pure-shear DEA, where the phase of the voltage signal is shifted by 180°. Photographs show the DEA in states 1 and 3. (*B*) Experimentally prescribed and measured state variables as a function of time, shown for the actuation conditions with the highest measured efficiency of 62.9% (Φ_p_ = 7 kV, *F*_p_ = 6 N). (*C*) The corresponding work cycle on the voltage–charge and the force–position planes. The enclosed areas correspond to the electrical energy input and the mechanical work output. (*D*) Measured electrical energy input, mechanical work output and (*E*) efficiency as a function of prescribed voltage and prescribed force. The DEA experienced dielectric failure at 8 kV. All data were measured with a cycle frequency of 0.25 Hz and a preload force *F*_preload_ of 2 N.

To showcase the generalizability of our method to other soft electrostatic actuators, we measured the efficiency of a DEA made from a silicone elastomer film (Elastosil 2030, Wacker) film under pure-shear deformation (12, 59) (see *Materials and Methods* for details on fabrication and experimental setup). While a Peano-HASEL actuator contracts when activated with voltage, a pure-shear DEA elongates; therefore, the voltage signal for the cycle used for the DEA is 180 degrees phase shifted (Fig. 7*A* and Movie S4). In the initial state (state 1), no voltage is applied to the actuator (Φ_1_ = 0), and it has zero charge (*Q*_1_ = 0). A preload force of *F*_1_ = 2 N is prescribed to stretch the actuator by 125%, so we therefore represent displacement instead of position, defining displacement as zero (*x*_1_ = 0) in state 1. From state 1 to state 2, the voltage is linearly increased to a voltage Φ_2_ = Φ_p_, and the actuator deforms to a displacement *x*_2_, and the charge on the electrodes is increased to *Q*_2_. From state 2 to state 3, the force is ramped up linearly, and the actuator further elongates to a displacement *x*_3_, and more charge is deposited onto the electrodes until the total charge reaches *Q*_3_. From state 3 to state 4, the voltage is reduced while the force is held constant. This leads to a decrease in charge to *Q*_4_ = 0, and the actuator contracts to a displacement *x*_4_. From state 4 to state 1, the force is reduced back to the preload force *F*_1_ = 2 N and the actuator returns to approximately its initial position *x*_1_ = 0. The prescribed and measured values are shown in Fig. 7*B*.

We used work-conjugate planes shown in Fig. 7*C* to calculate the electrical energy input and the mechanical work output as a function of prescribed peak voltages Φ_p_ ranging from 4 to 7 kV (beyond which, dielectric breakdown occurred), and as a function of prescribed peak forces *F*_p_ ranging from 2.1 N to 9 N. Both the measured electrical energy input and mechanical work output increased with both the prescribed peak voltage and with load (Fig. 7*D*), and a maximum efficiency of 62.9% is observed at 6 N of peak force, and 7 kV of peak voltage, which is the highest efficiency measured for DEAs using closed work cycles (Fig. 7*E*) (39, 60).

## Discussion

The framework presented in this article offers a robust and comprehensive method for evaluating the energy conversion efficiency of soft electrostatic actuators by analyzing closed work cycles on voltage–charge and force–position planes. Using the Peano-HASEL as a model system, we demonstrate that the efficiency of electrostatic actuators is highly dependent on operating parameters such as load, driving voltage, and frequency; for the specific cycle, we have chosen to analyze, we measured a maximum efficiency of 63.6%—considerably higher than the previous value of 21% we reported for HASEL actuators (28), where only one combination of load and voltage was considered. Therefore, parametric studies are essential to comprehensively characterize the efficiency of electrostatic actuators, and to identify operating conditions that maximize efficiency. Given these insights, our work identifies two concurrent approaches to enhance efficiency for a given load: i) using the optimum voltage for each load; ii) tuning the width of the actuator to match the load. Additionally, through system-level design, such as stacking actuators in parallel and in series (55), and adding mechanical transmission elements (e.g., lever arms), the stroke and force of an actuator can be tuned to operate under the most efficient conditions. Furthermore, for varying loads, one could use stacks of parallel actuators that can be electrically addressed individually; here, for higher loads a higher number of individual actuators would be activated in order to operate near peak efficiency.

We further studied the effects of cycle frequency on efficiency, which revealed that at very low frequencies, the electrical work input is larger due to charge leakage. We also showed that interfacial charge accumulation affects actuator efficiency, which is dependent on the dielectric materials combination. Additionally, we designed experiments to analyze inherent electrical and mechanical losses, which provide a qualitative measure of the degree of irreversibility of each process in the analyzed cycle, thereby allowing one to appraise the efficiency of different types of actuators and to preselect materials that decrease losses.

It is important to note that while we achieved a measured efficiency of 63.6%, this still leaves parts of the 36.4% of energy losses unexplained. In addition to charge leakage through the dielectric layers and interfacial charge accumulation, as mentioned above, loss mechanisms could include inelastic buckling of the films, friction effects, dielectric hysteresis, Joule heating of the electrodes, or other mechanisms. We have suggested the analysis of inherent mechanical and electrical losses as a possible tool to quantify losses; the specific loss mechanisms and their respective impact on efficiency should be thoroughly investigated in future studies. Additionally, our system only measures charge flows on the ground side of the soft electrostatic actuator, meaning it is not capable of measuring corona losses to the environment on the high voltage side, which would reduce the efficiency. Electrical circuitry could be designed to characterize these corona losses on the high voltage side; however, encapsulation of the electrodes is likely to substantially reduce or eliminate such corona losses. Our measurement cycle assumes that charge is recovered during actuator discharge, which means that an effective charge recovery method is needed to achieve the reported efficiency values in practice. Furthermore, although the specific closed cycle we have used to analyze actuators provides a systematic and intuitive method for quantifying efficiency, we do not claim that it is the best cycle in terms of maximizing efficiency – other cycles may result in higher efficiencies and the selection of such cycles warrants future study.

Finally, to showcase the generalizability of our setup across different electrostatic actuators, we measured the efficiency of a DEA under pure-shear deformation, and a maximum efficiency of 62.9% was observed.

In conclusion, the presented comprehensive method to study the fundamental mechanisms of electrical-to-mechanical energy conversion facilitates the systematic characterization of the efficiency of soft electrostatic actuators, thereby allowing in-depth comparison between different technologies and facilitating the development of the next generation of highly efficient soft robots.

## Materials and Methods

Peano-HASELs were fabricated according to Mitchel et al. (25). The liquid dielectric used is 5-cSt silicone oil (Carl-Roth CAS: 63148-62-9). The electrodes are made from screen-printable carbon ink (CI-2051, Nagase ChemteX America LLC.). Unless otherwise stated, the solid dielectric film used is 15-μm Mylar polyester film (Petroplast Mylar 850). Additional solid dielectric films are 20-μm L0WS polyester film (Multi-Plastics HSF4000W Clear Heat Sealable (Weld Seal) Polyester Film), 20-μm BOPP (biaxially oriented polypropylene) (Multi-Plastics HSF5114H Clear Heat Sealable Polypropylene Film), and 16-μm PLA (polylactic acid) (Pütz GmbH + Co. Foilen KG Nativia NTSS).

To fabricate the DEA, a rectangle of ready-made elastomer film is adhered to a rigid mount without prestretching and electrodes are painted onto both sides. The solid dielectric film is 100-µm ELASTOSIL^®^ Film 2030 250/100 (Wacker) (relative permittivity of 2.8). The electrodes are made from conductive carbon grease (MG Chemicals 846).

See the *SI Appendix* for the more details on the fabrication methods for both actuators.

## Supplementary Material

Appendix 01 (PDF)

Movie S1. Analysis of closed-cycle energy efficiency for a Peano-HASEL actuator.

Movie S2. Measurement of inherent mechanical loss.

Movie S3. Measurement of inherent electrical loss.

Movie S4. Analysis of closed-cycle energy efficiency for a pure-shear dielectric elastomer actuator.

## Data Availability

All study data are included in the article and/or supporting information.
