# Supplementary material for: A method to study and enhance the energy efficiency of soft electrostatic actuators
Source: Proc Natl Acad Sci U S A. 2026 Feb 6;123(6):e2527676123. doi: 10.1073/pnas.2527676123 (PMC12890970; doi:10.1073/pnas.2527676123)
Supplement: Supplementary file 1 — Appendix 01 (PDF) [file pnas.2527676123.sapp.pdf]

PNAS

Supporting Information for

A method to study and enhance the energy efficiency of soft electrostatic actuators

Steven L. Zhang,[a] Toshihiko Fukushima,[a] Sophie Kirkman,[a] Soo Jin Adrian Koh,[a] Philipp Rothemund,[a,b]* Christoph Keplinger[a,c,d]*

[a]Robotic Materials Department, Max Planck Institute for Intelligent Systems, Heisenbergstraße 3, 70569 Stuttgart, Germany

[b]Institute for Adaptive Mechanical Systems, University of Stuttgart, Heisenbergstraße 3, 70569 Stuttgart, Germany

[c]Paul M. Rady Department of Mechanical Engineering, University of Colorado Boulder, Boulder, CO 80309, USA.

[d]Materials Science and Engineering Program, University of Colorado Boulder, Boulder, CO 80309, USA.

Email: *philipp.rothemund@iams.uni-stuttgart.de, ck@is.mpg.de

This PDF file includes:

Supporting text

Figures S1 to S13

Legends for Movies S1 to S4

Other supporting materials for this manuscript include the following:

Movies S1 to S4

Supporting Information Text

Fabrication of 7-pouch Peano-HASEL actuator

The Peano-HASEL actuators were fabricated following basic principles outlined in Mitchell et al.(*1*): First, two solid polymer films were sealed together with a CNC heat sealer into seven rectangular pouches, each with a width of 6 cm and length of 2 cm. Then, electrodes of width 6 cm and length 1 cm were screen printed onto both sides of the heat-sealed films. Each pouch was filled with 1.8 mL of silicone oil. Then, copper tape (3M, Scotch 1245) was attached to the electrode to connect the actuator to the electrical circuit. Finally, mounts were laser cut from acrylic plates (2 mm thickness) and bonded chemically to the actuators with a few drops of chloroform ($CHCl_3$, Carl Roth CAS: 67-66-3). Mechanical pressure is applied to spread the chloroform evenly between the layers. The chloroform acts as a solvent, temporarily dissolving surface molecules of both the acrylic and film. As the chloroform evaporates, the dissolved polymers re-solidify, creating a strong bond.

Fabrication of single-pouch Peano-HASEL actuator

The single pouch Peano-HASEL actuator was fabricated in the same way as a 7-pouch Peano-HASEL actuator; instead of seven pouches for heat sealing and printing, only a single pouch was used.

Details on experimental setup

The top mount of the actuator was screwed onto an acrylic glass stand. The bottom mount was screwed onto the lever arm of a dual mode muscle lever system (310-LR, Aurora Scientific 3000), as shown in Figure 2e. One electrode was connected to a high voltage amplifier (Trek 610E), which acts as a power source for the actuator. The other electrode was connected to an electrometer (Keithley 6514) which is protected in case of dielectric breakdown of the actuator by a fuse (30 mA, fast response) in series with the Keithley to prevent overcurrents and a bi-directional transient-voltage-suppression (TVS) diode (Model 1.5KE56CA-TP, Micro Commercial Components) in parallel to prevent overvoltages (circuit diagram shown in Figure 2d). The Keithley, the protection circuit, and the Trek were all connected to earth ground, which acts as a common reference point for electric potential (Figure 2e).

Measurement of efficiency and inherent losses of a Peano-HASEL actuator

The output voltage and force signals were generated by a personal computer (Precision 3660, Dell) using programming software (MATLAB R2023a, MathWorks) or LabView (2022 Q3 64-bit, National Instruments) and sent to the high voltage amplifier and to the dual mode muscle lever system via a data acquisition system (DAQ; USB 6212, National Instruments). The data acquisition system reads the signal of the high voltage amplifier via the low voltage replica channel, charge via the output voltage from the electrometer, and force and position via output voltages from the dual mode muscle lever system. To zero the measurement, the value of the first data point was subtracted from all subsequent data points of charge and position.

Prescribing charge and position signal

In addition to prescribing voltage and force signals, charge and position signals can also be prescribed. When prescribing charge, the high voltage amplifier regulates the current flow to the actuator. Since charge is the first order integration of current with respect to time ($Q = \int I \, \mathrm{d}t$), a constant current will result in a linear ramping up of charge. To maintain a constant charge, the amplifier regulates the current flow to be zero. For position control, the muscle lever system regulates and prescribes the position and measures the force applied on the lever system.

Modified prescribed signal for high frequency cycles

To achieve reliable measurements in the high frequency cycles shown in Figure S9, the cycles presented in Figure 3b were modified. Position was prescribed instead of force on the dual mode lever system to

achieve higher frequency motion. To avoid sharp edges between the ramp and holding periods, the linear ramps connecting the initial and peak positions were replaced with sinusoid ramps of the same time duration and with a tangent of 0 degrees at the connection point to the holding segment.

Fabrication of pure-shear DEA

The dielectric elastomer actuator was designed to operate under pure-shear deformation. The dielectric material used was a ready-made silicone film (ELASTOSIL 2030 250/100, Wacker) with an initial thickness of 100 µm. With no lateral prestretch ($\lambda_2 = 1$), a rectangle of elastomer (12 cm by 5.5 cm) was mounted onto two laser-cut acrylic mounts with Sil-Poxy silicone adhesive (Smooth-On). The adhesive was allowed to cure for 6 hours at room temperature.

The active area of the elastomer had a width of 8 cm and a length of 2 cm. Both sides of the active area of the elastomer were coated with carbon grease (MG Chemicals) using a paintbrush. Copper tape was used to connect one electrode to high voltage and the other to ground.

Measurement of efficiency of DEA

The output voltage and force signals were generated by a personal computer (Precision 3660, Dell) using programming software (MATLAB R2023a, MathWorks) and sent to the high voltage amplifier and to the dual mode muscle lever system via a data acquisition system (DAQ; USB 6212, National Instruments). The data acquisition system reads the signal of the high voltage amplifier via the low voltage replica channel, charge via the output voltage from the electrometer, and force and position via output voltages from the dual mode muscle lever system. A preload of 2 N is applied onto the DEA, causing the DEA to vertically stretch to a length of 1.25 times that of its original length ($\lambda_1 = 1.25$). To zero the measurement, the value of the first data point was subtracted from all subsequent data points of charge and position. Since there is a prestretch on the DEA, we labeled the position as a displacement.

Recording of movies

All movies of Peano HASEL actuators were recorded using a DLSR camera (Canon R5) with lens (Canon RF 24-70 mm f/2.8L IS USM). The movie of the dielectric elastomer actuator was recorded using a DLSR camera (Canon R5) with lens (Canon RF 100 mm f/2.8 L Macro IS USM).

Figures

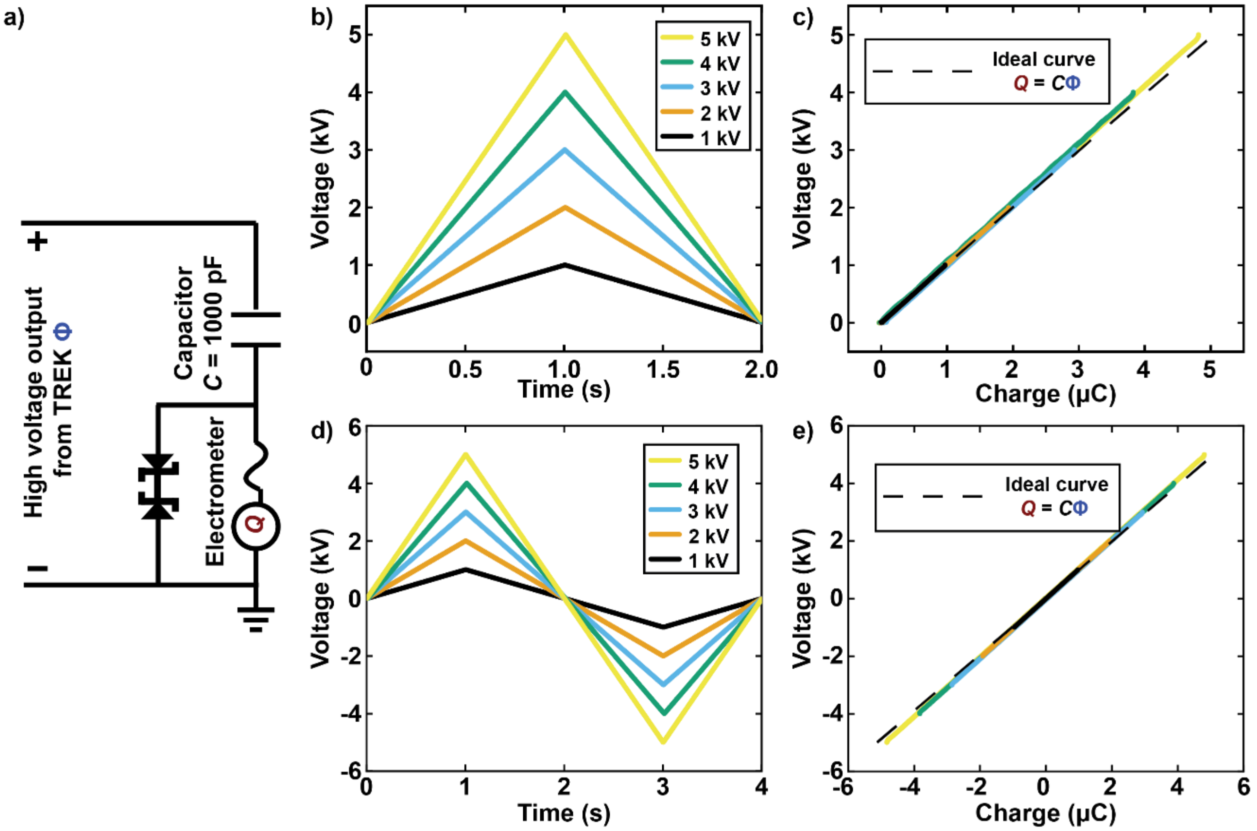

Figure S1. Evaluation of accuracy of the electrometer used for efficiency measurement. a) Circuit diagram of the experimental setup. A high voltage is applied to a 1000 pF capacitor. b) Prescribed single polarity voltage signal with respect to time applied across the capacitor and c) the corresponding voltage-charge curves. The measured curves are close to the voltage-charge curve of an ideal capacitor, where $Q=C\Phi$, with C = 1000 pF. d) Prescribed reversing polarity voltage signal with respect to time applied across the 1000 pF capacitor, and e) the corresponding voltage-charge curves. The measured curves are close to the voltage-charge curve of an ideal 1000 pF capacitor.

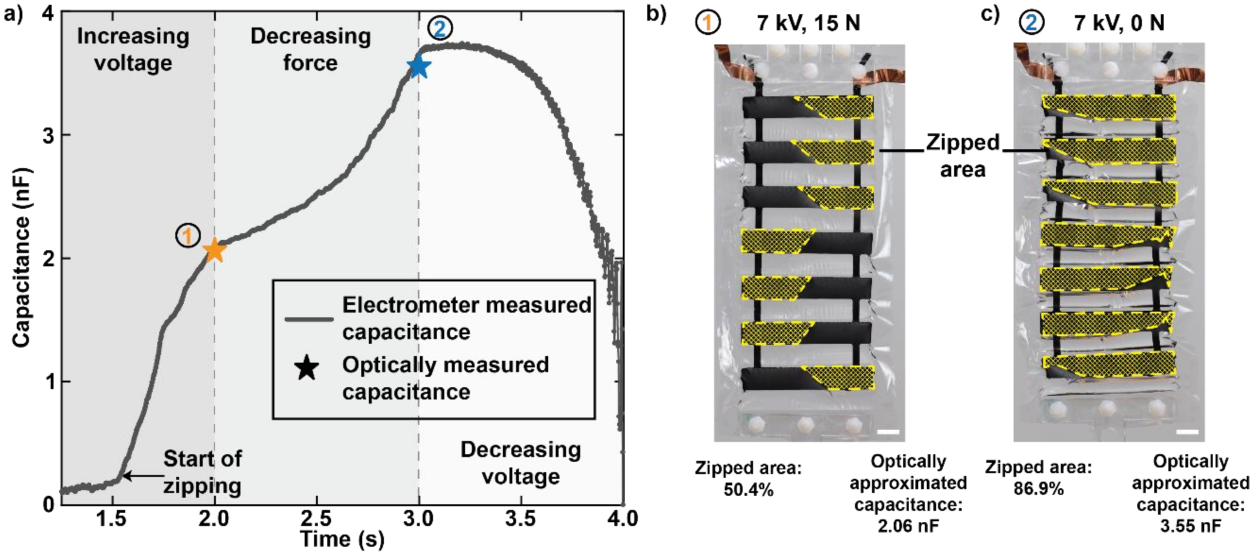

Figure S2. Evaluating the accuracy of the electrometer in measuring capacitance of Peano-HASEL actuators. a) Measured capacitance as a function of time during an efficiency-measurement cycle. Star markers show the optically measured capacitance at two selected times as calculated from b) and c), which shows close alignment to the capacitance measured by the electrometer. b) Photograph of a Peano-HASEL actuator at 7 kV and an applied force of 15 N (point 1 of (a)) (scale bar: 10 mm). The zipped area A_{zip} was calculated using image analysis of the photograph (ImageJ) and capacitance was calculated by using $C = \varepsilon_r \varepsilon_0 A_{zip}/2t$, where ε_r is relative permittivity of the Mylar film material (3.3), ε_0 is the permittivity of free space ($8.85\text{x}10^{-12}$ F/m), and t is the thickness of the Mylar (15 µm). This capacitance is the optically estimated capacitance (2.06 nF). We calculate the capacitance using two assumptions: 1) There is no oil in the zipped area, and 2) the capacitance of the unzipped area can be neglected. c) Photograph of Peano-HASEL actuator at 7 kV with applied force of 0 N (point 2 of (a)) (scale bar: 10 mm). The capacitance of the zipped area is optimally estimated to be 3.55 nF.

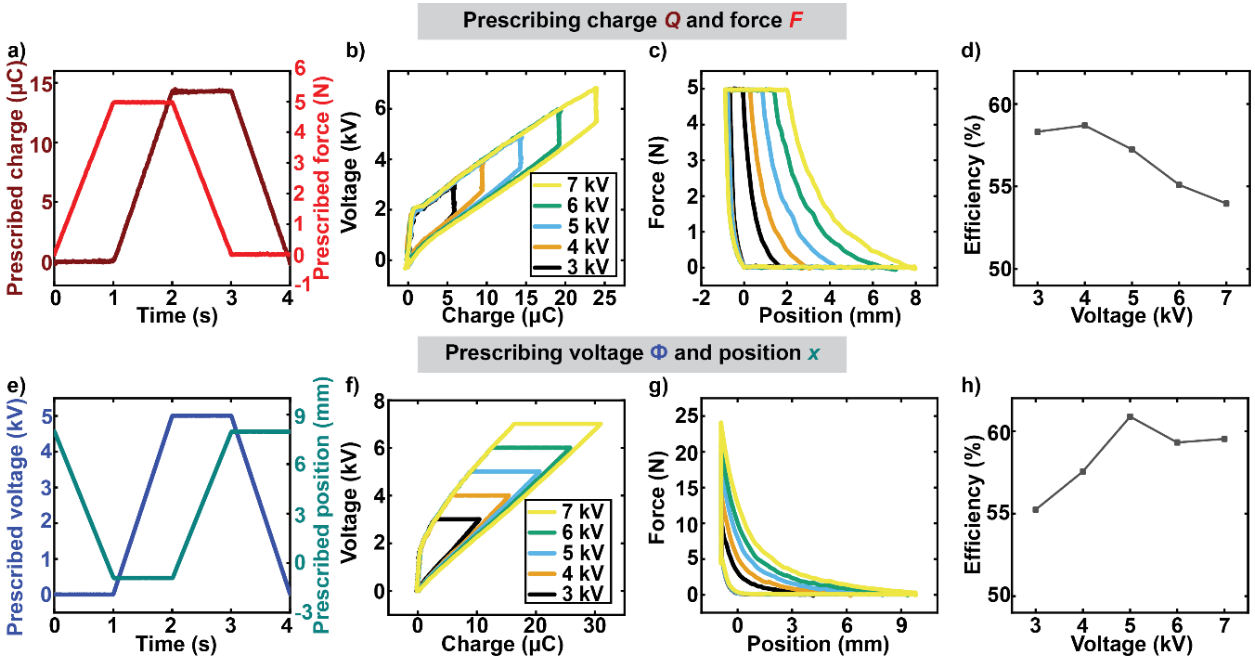

Figure S3. Closed-cycle efficiency measurement prescribing charge and force, and prescribing voltage and position. a) Experimentally prescribed charge and force for closed-cycle efficiency measurement. The corresponding work cycle on b) the charge-voltage plane and c) the force-position plane. d) Electromechanical conversion efficiency as a function of voltage reached by prescribing charge and force. e) Experimentally prescribed voltage and position for closed cycle efficiency measurement. The corresponding work cycle on f) the charge-voltage plane and g) the force-position plane. h) Electromechanical conversion efficiency as a function of voltage reached by prescribing position and voltage.

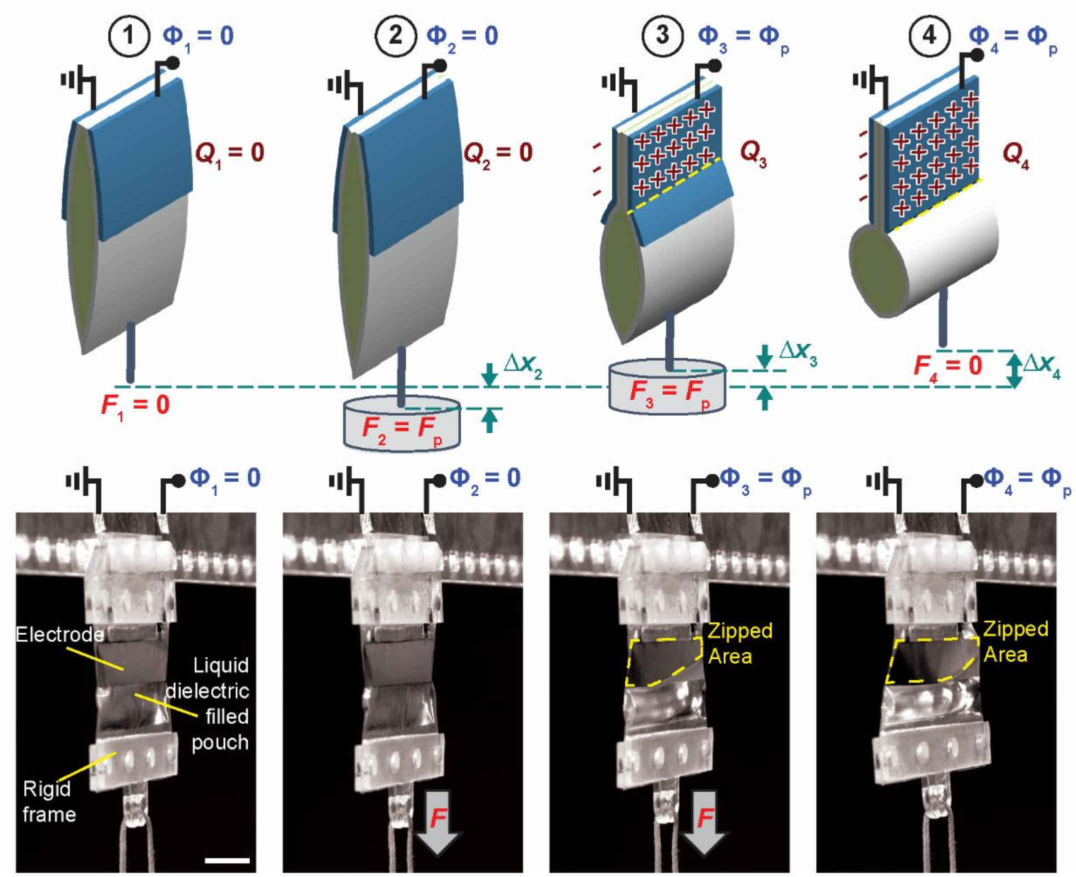

Figure S4. Sketches and photographs of a single-pouch Peano-HASEL actuator to illustrate the four states of the efficiency-measurement cycle. Left to right 1) voltage off, force off 2) voltage off, force on 3) voltage on, force on 4) voltage on, force off (scale bar: 20 mm). This cycle is referenced in **Figure 3a.**

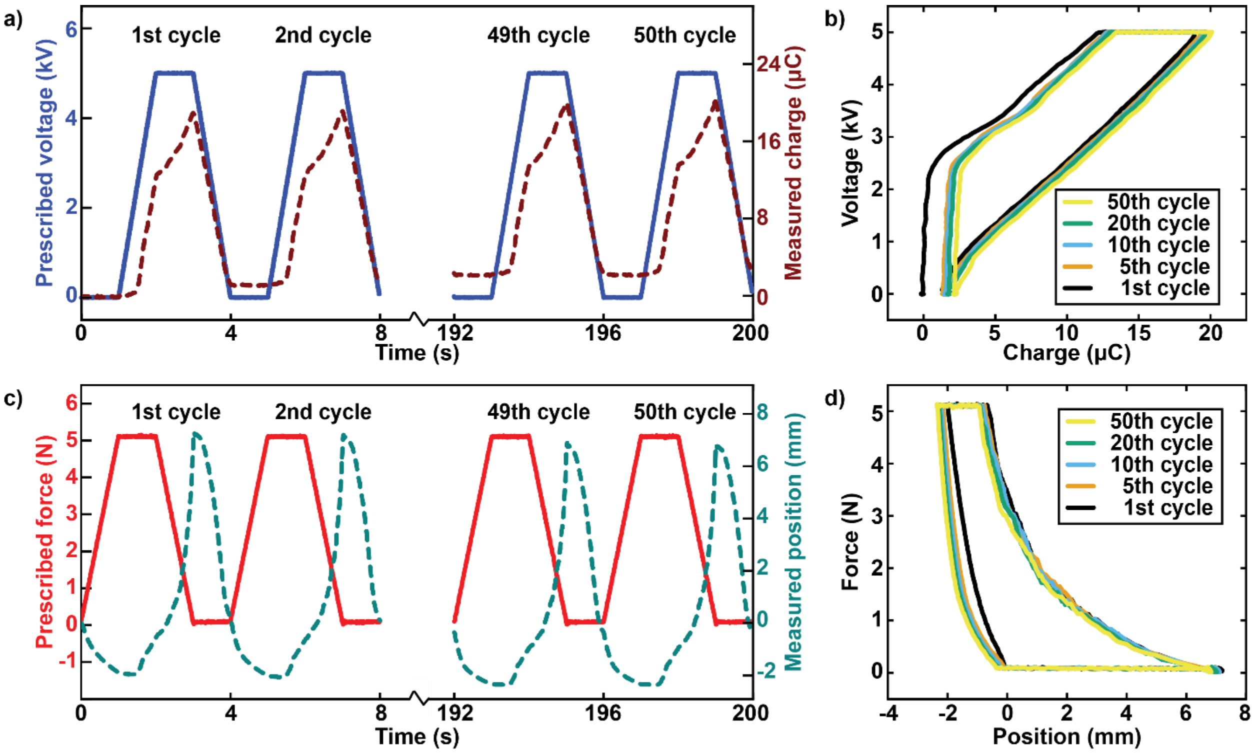

Figure S5. Efficiency measurement in steady-state conditions. a) Prescribed voltage and measured charge with respect to time for multiple efficiency-measurement cycles. b) Corresponding voltage-charge plots of selected cycles. We run 10 cycles before starting to record data in order to ensure steady-state is attained. c) Prescribed force and measured position with respect to time for multiple efficiency cycles, and d) the corresponding force-position curves comparing the same selected cycles as in (b). Similar to the voltage-charge cycles, also force-position reaches steady-state.

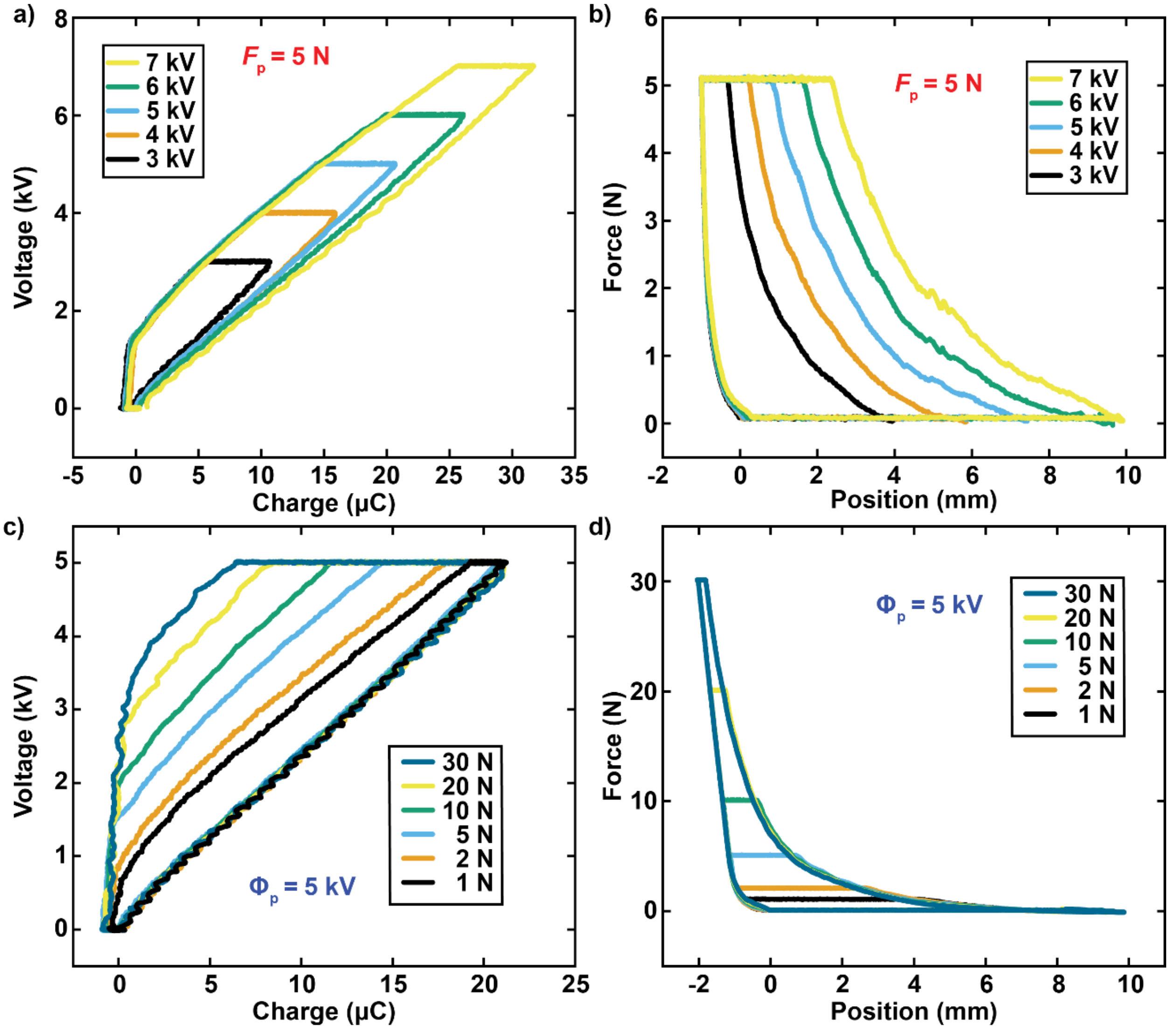

Figure S6. Closed-cycle efficiency measurement for Peano-HASEL actuators varying the maximum applied voltage and the maximum applied force. a) Prescribed voltage plotted as a function of the measured charge and b) prescribed force as a function of the measured position at different peak voltages Φ_p at the same peak applied force F_p of 5 N. c) Prescribed voltage plotted as a function of the measured charge and d) prescribed force as a function of the measured position at different peak forces F_p at the same peak voltage Φ_p of 5 kV.

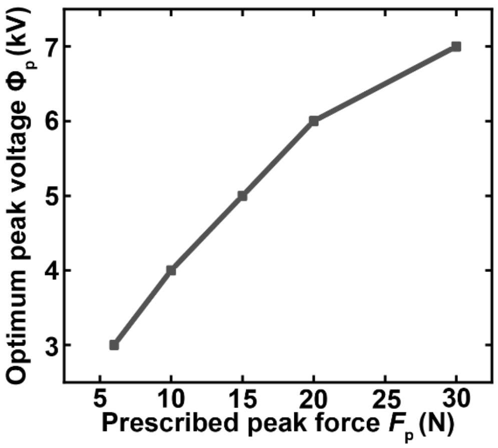

Figure S7. For a given force, there is an optimum peak voltage Φ_p which results in the highest efficiency.

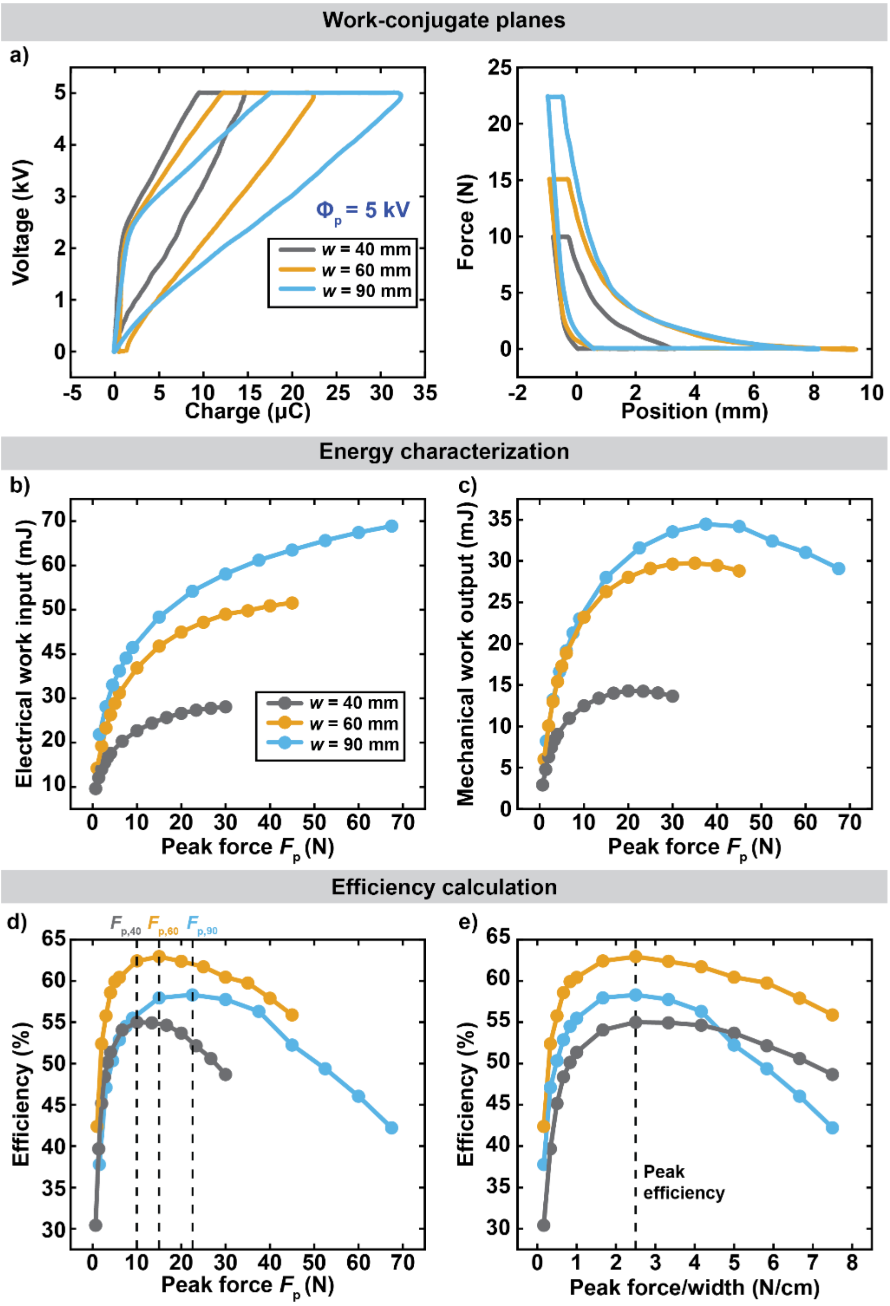

Figure S8. Effect of actuator width on efficiency. a) The work cycles on the voltage-charge and force-position planes for actuators of widths of 40 mm and 90 mm compared with the 60-mm-wide actuator, which is used in all other experiments. The applied peak voltage is 5 kV. b) Measured electrical work input and c) measured mechanical work output as a function of the prescribed force. d) Electromechanical conversion efficiency as a function of prescribed force and e) as a function of normalized force. The normalization shows the peak efficiency occurs at the same force/width (2.5 N/cm). We note that the maximum overall value for efficiency is reached with a medium width actuator; we speculate that the somewhat lower efficiency for the narrow actuator stems from side constraints (buckling of the film), while the somewhat lower efficiency for the wide actuator stems from inhomogeneous zipping.

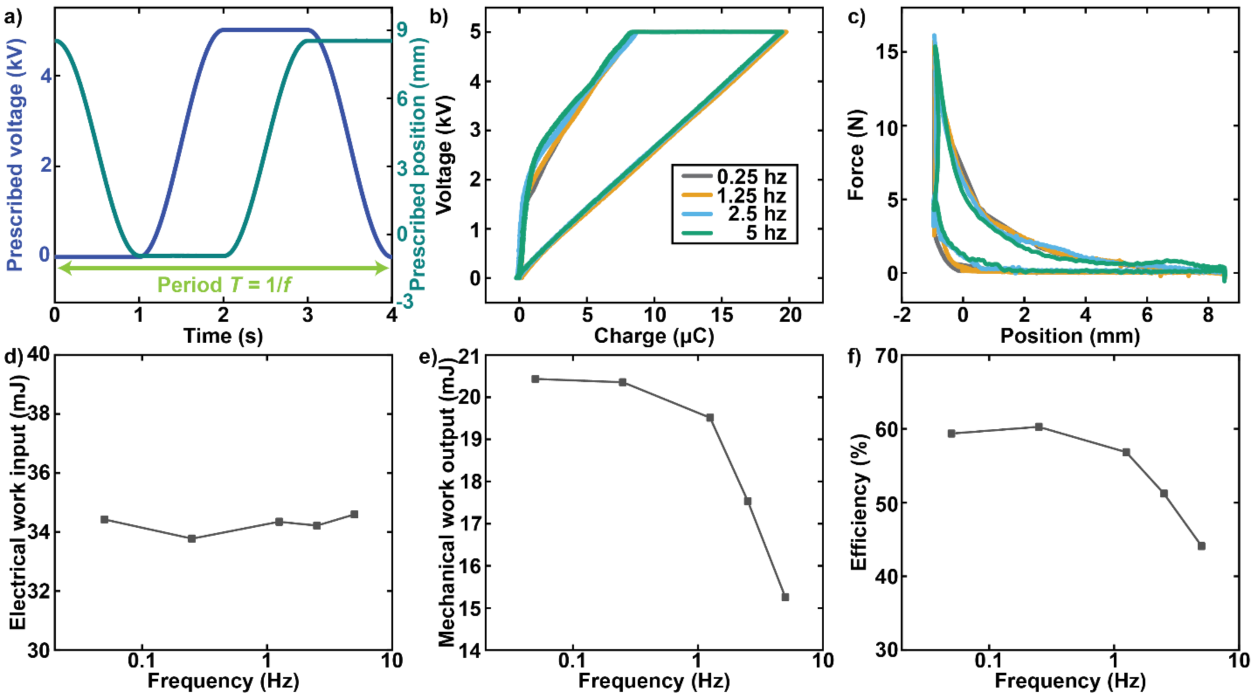

Figure S9. Position control to obtain higher-frequency cycles. a) Prescribed voltage and position as a function of a time, using rounded corners with a sinusoidal ramp instead of a linear ramp. b) Voltage-charge work cycles measured at different frequencies. c) Force-position work cycles at different frequencies. d) Electrical work input and e) mechanical work output as a function of frequency. f) The resulting electrical to mechanical conversion efficiency.

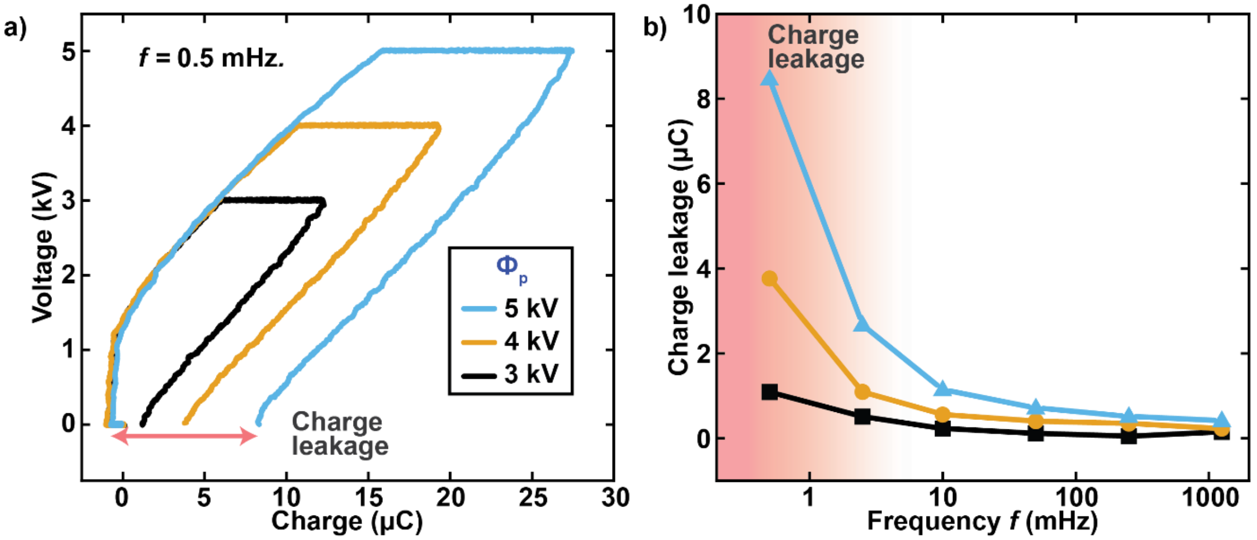

Figure S10. Quantification of charge leakage. a) Prescribed voltage plotted as a function of the measured charge at different peak voltages at the same peak force of 5 N at a frequency of 0.5 mHz. The difference in charge at 0 kV corresponds to the charge leaked during the cycle. b) Charge leakage as a function of frequency at peak voltages 3 kV, 4 kV, and 5 kV and peak force of 5 N.

a)

Material	Thickness (µm)
BOPP	20
PLA	16
Mylar	15
L0Ws	20

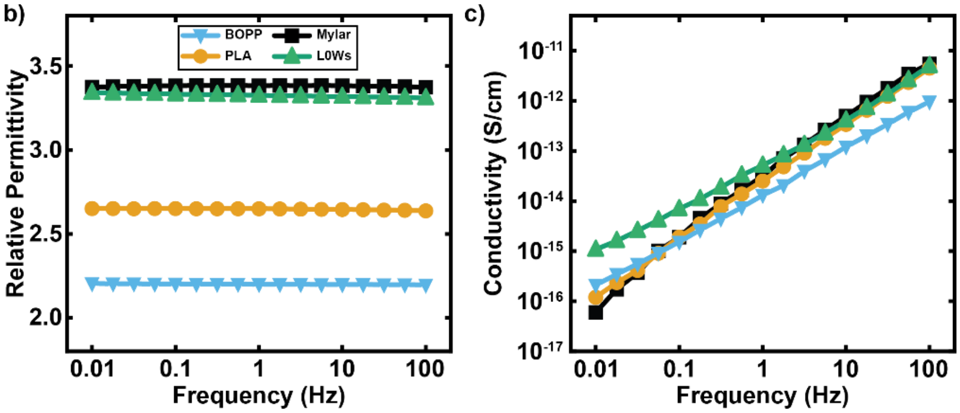

Figure S11. Dielectric spectroscopy measurement of different films using a Novocontrol Broadband Dielectric Spectrometer with HVB4000 Test Interface. a) Thicknesses of the characterized films, which include BOPP (biaxially oriented polypropylene), PLA (polylactic acid), Mylar (polyethylene terephthalate) and L0Ws (polyethylene terephthalate). b) Measured relative permittivity and c) measured conductivity as a function of frequency at an applied rms-voltage of 500 V. Circular silver electrodes with diameter of 23 mm and thickness of 200 nm were evaporated onto both sides of each dielectric film.

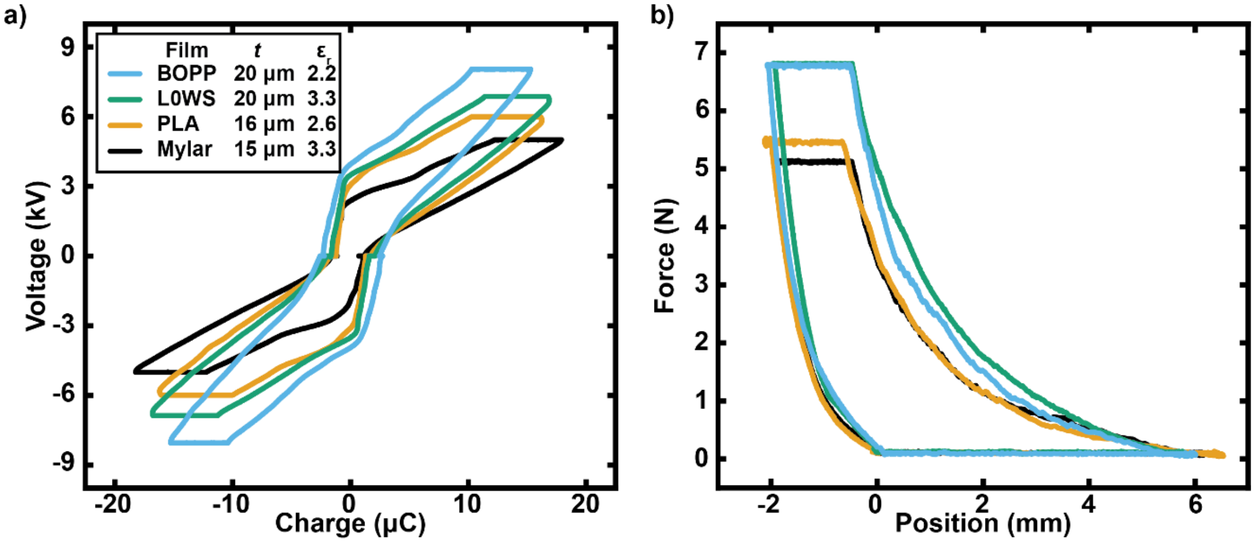

Figure S12. Characterization of effect of different films on work-conjugate planes. a) Voltage-charge and b) force-position planes of actuators with different films.

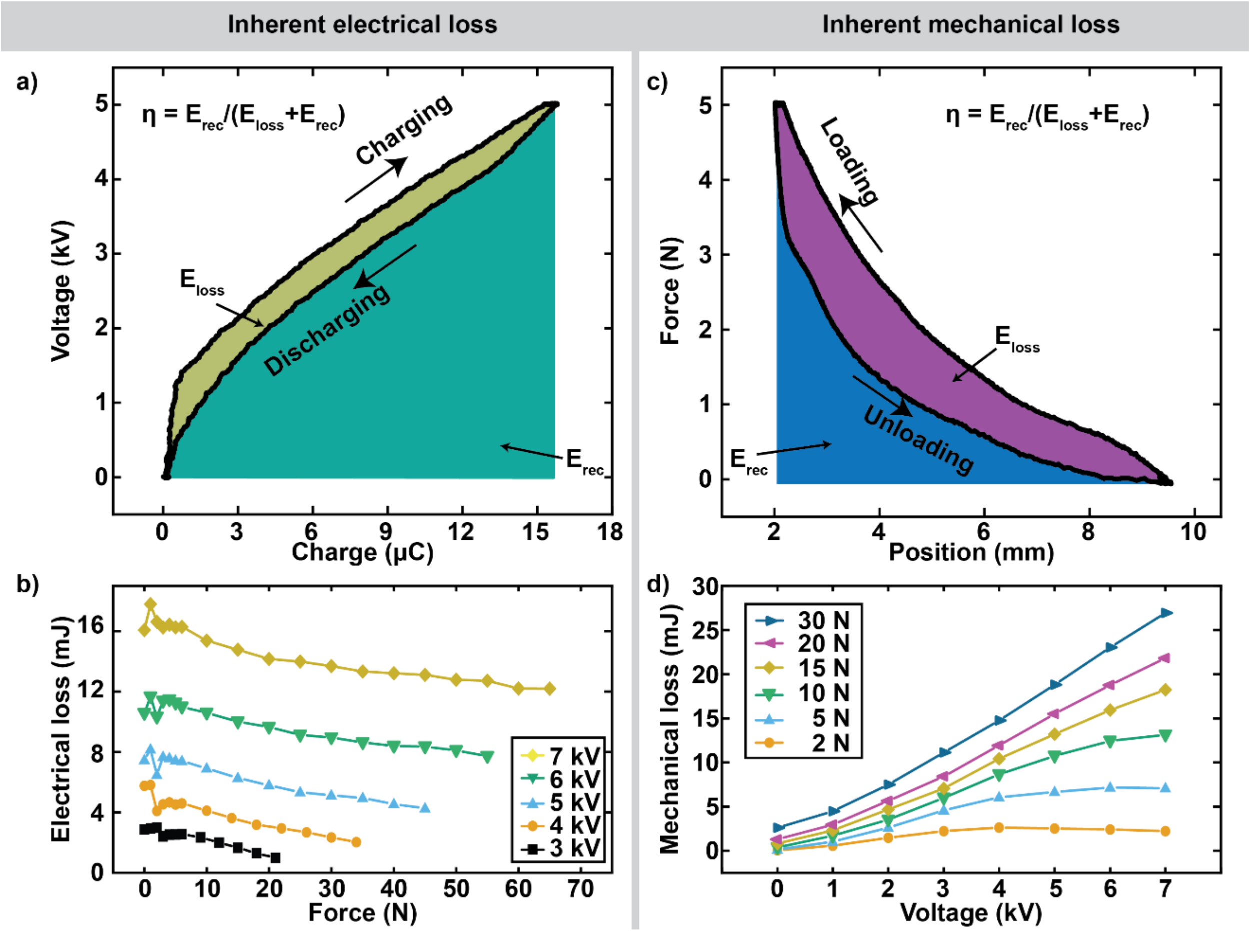

Figure S13. Calculation of charge-discharge efficiency and load-unload efficiency and inherent electrical and inherent mechanical loss. a) Calculation of the charge-discharge efficiency for Figure 6e. $E_{loss,elec}$ is the inherent electrical loss during one charging and discharging cycle. $E_{rec,elec}$ is the electrical energy recovered during discharging. b) Measured inherent electrical loss as a function of force at different applied peak voltages. c) Calculation of load-unloading efficiency for Figure 6j, $E_{loss,mech}$ is the inherent mechanical loss during one loading and unloading cycle. $E_{rec,mech}$ is the mechanical energy recovered during unloading. d) Measured inherent mechanical loss as a function of voltage and at different applied peak forces.

Movie S1. Analysis of closed-cycle energy efficiency for a Peano-HASEL actuator.
Movie S2. Measurement of inherent mechanical loss.
Movie S3. Measurement of inherent electrical loss.
Movie S4. Analysis of closed-cycle energy efficiency for a pure-shear dielectric elastomer actuator.

References

1. S. K. Mitchell *et al.*, An easy-to-implement toolkit to create versatile and high-performance HASEL actuators for untethered soft robots. *Advanced Science* **6**, 1900178 (2019).
